# Biomass for thermochemical conversion: targets and challenges

**DOI:** 10.3389/fpls.2013.00218

**Published:** 2013-07-01

**Authors:** Paul Tanger, John L. Field, Courtney E. Jahn, Morgan W. DeFoort, Jan E. Leach

**Affiliations:** ^1^Bioagricultural Sciences and Pest Management, Colorado State UniversityFort Collins, CO, USA; ^2^Engines and Energy Conversion Laboratory, Department of Mechanical Engineering, Colorado State UniversityFort Collins, CO, USA; ^3^Natural Resource Ecology Laboratory, Colorado State UniversityFort Collins, CO, USA

**Keywords:** biomass composition, thermochemical conversion, high-throughput phenotyping, silica, moisture content, proximate/ultimate analysis, heating value

## Abstract

Bioenergy will be one component of a suite of alternatives to fossil fuels. Effective conversion of biomass to energy will require the careful pairing of advanced conversion technologies with biomass feedstocks optimized for the purpose. Lignocellulosic biomass can be converted to useful energy products via two distinct pathways: enzymatic or thermochemical conversion. The thermochemical pathways are reviewed and potential biotechnology or breeding targets to improve feedstocks for pyrolysis, gasification, and combustion are identified. Biomass traits influencing the effectiveness of the thermochemical process (cell wall composition, mineral and moisture content) differ from those important for enzymatic conversion and so properties are discussed in the language of biologists (biochemical analysis) as well as that of engineers (proximate and ultimate analysis). We discuss the genetic control, potential environmental influence, and consequences of modification of these traits. Improving feedstocks for thermochemical conversion can be accomplished by the optimization of lignin levels, and the reduction of ash and moisture content. We suggest that ultimate analysis and associated properties such as H:C, O:C, and heating value might be more amenable than traditional biochemical analysis to the high-throughput necessary for the phenotyping of large plant populations. Expanding our knowledge of these biomass traits will play a critical role in the utilization of biomass for energy production globally, and add to our understanding of how plants tailor their composition with their environment.

## Introduction

### Multiple pathways from feedstock to energy

Our society and economy rely heavily on energy from fossil fuels. Most (84%) of the world's energy comes from fossil fuels and demand will increase as world energy consumption is expected to increase 53% by 2035 (EIA, 2011). As prices rise, unconventional fossil resources (tar sand oil, shale gas, arctic and deepwater oil) may become economically viable to extract, but they are ultimately a limited resource and carry risks to our health and environment (Kelly et al., 2010; Osborn et al., 2011; Frohlich, 2012).

Bioenergy, derived from plants that use sunlight and CO_2_ to assimilate carbon into biomass, has emerged as a potentially sustainable energy source with low climate impact. The Renewable Fuel Standard, enacted in 2005 and expanded in 2007, mandates liquid biofuel production in the US (EISA, 2007). The majority of the fuel produced today to support this mandate is derived from either ethanol fermented from corn grain, or biodiesel from soybean oil, but by the year 2022, 58% of the legislated 36 billion gallons is required to be produced from cellulosic or advanced cellulosic biomass. Technological advances and commercialization have not occurred as quickly as expected, and several barriers must be overcome to achieve these targets (National Research Council, 2011).

One of these barriers is the production of high quality biomass that can be economically converted into useful energy products. Biomass quality depends on the plant composition—cellulosic biomass is primarily comprised of cellulose, hemicellulose, lignin, and lesser amounts of other extractable components such as pectins, proteins, etc. that make up the plant cell wall. Cellulose is a polymer of D-glucose. Hemicellulose is a general term for heterogeneous branched five and six carbon sugars. Lignin is a complex branched polymer of phenolics, and is classified as three major types, based on the monomers present: sinapyl (S) coumaryl (H) and coniferyl (G) (Albersheim et al., 2010). The proportions and specific chemical composition of these components varies greatly among species (Pauly and Keegstra, 2008; Carroll and Somerville, 2009; Allison et al., 2010; Tao et al., 2012b; Zhao et al., 2012a). Furthermore, significant compositional variation has been observed within a species (Jin and Chen, 2006; Tao et al., 2012b), within tissue type (Summers et al., 2003; Monti et al., 2008; Rancour et al., 2012; Sabatier et al., 2012), as well as between developmental stages (Rancour et al., 2012), cell types, and even regions of the cell wall (Albersheim et al., 2010). Additional variability is observed throughout the growing season and as plants senesce (Landström et al., 1996; Adler et al., 2006; Hodgson et al., 2010; Nassi o Di Nasso et al., 2010; Singh et al., 2012; Zhao et al., 2012b), as well as across different environments (Adler et al., 2006; Mann et al., 2009; Allison et al., 2011; Monono et al., 2013; Serapiglia et al., 2013).

Variation, either naturally existing variation or driven with biotechnology, is the ultimate source of improved crop varieties. Most feedstock improvement efforts have focused on the enzymatic conversion pathway, and how to increase the availability of components of plant biomass that can readily be converted into simple sugars and fermented into alcohols; i.e., maximizing cellulose and minimizing lignin. Other articles in this research topic address challenges and advances in enzymatic conversion, as have multiple recent reviews (Vermerris, 2011; Feltus and Vandenbrink, 2012; Jordan et al., 2012; Nookaraju et al., 2013).

**Figure 1:**
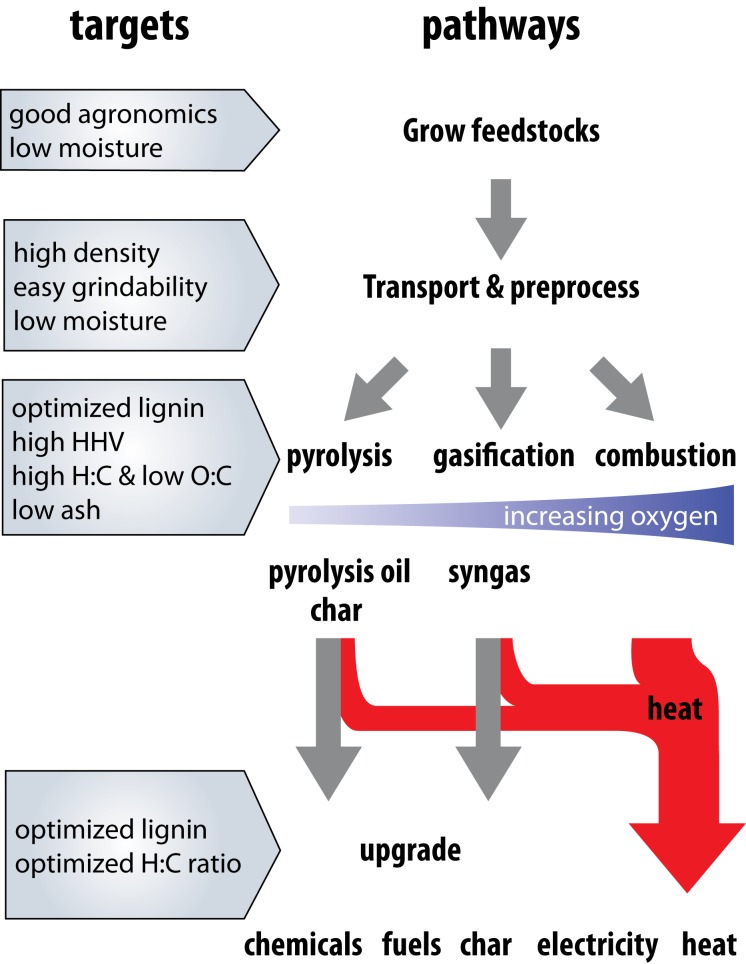
**Overview of the steps involved in growing, transporting, processing, and converting biomass into thermochemical energy products**. Pyrolysis, gasification, and combustion take place under conditions of increasing oxygen availability during the reactions. Particle residence time and temperature may be optimized to yield different proportions and types of products. Boxes represent the properties important for each step (growing, transport and processing, conversion, upgrading). The primary products of each process and the potential end uses are highlighted. Note that intermediate products such as syngas and pyrolysis oil can be upgraded to chemicals or liquid transportation fuels or converted to obtain electricity and heat. Agronomic traits include those traits that allow the plant to survive and produce acceptable yields.

A promising alternative form of bioenergy production is via thermochemical conversion—the controlled heating or oxidation of biomass (Demirbas, 2004; Goyal et al., 2008). The term covers a range of technologies including pyrolysis, gasification, and combustion which can be configured to produce outputs of heat, electricity, or gaseous or liquid precursors for upgrading to liquid fuels or chemical feedstocks (Figure 1 and Butler et al., 2011; Wang et al., 2011; Brar et al., 2012; Bridgwater, 2012; Solantausta et al., 2012). Thermochemical technologies show great promise for the production of renewable electricity, both in the context of biomass co-firing in existing coal powerplants (Demirbaş, 2003b; Baxter, 2005), and for decentralized electrification projects in developing countries (Yin et al., 2002; Hiloidhari and Baruah, 2011; Shackley et al., 2012). Thermochemical produced electricity could help fulfill standards enacted in many US states that require a certain percentage of electricity be produced from renewable sources (Carley, 2009; DOE DSIRE, 2012; EIA, 2012). In some cases, thermochemical production of renewable electricity or liquid fuels and associated co-products is the most effective use of biomass for fossil energy displacement (Botha and von Blottnitz, 2006; Campbell et al., 2009; Cherubini et al., 2009; Searcy and Flynn, 2010; Giuntoli et al., 2012).

A well-functioning system requires the pairing of appropriate feedstocks and conversion technologies (Robbins et al., 2012), but optimization of biomass for thermochemical conversion has received little attention. The paradigm within which plant biologists discuss and analyze biomass is different than that of engineers analyzing feedstocks for thermochemical systems. While there is overlap between the paradigms, thermochemical feedstock development could focus on traits or approaches that provide the most direct path to optimized feedstock composition. In this review, we discuss how, through collaboration of biologists and engineers, optimized biomass composition and process engineering might result in reduced transport and pre-processing costs and maximized energy yields via thermochemical utilization of biomass.

We begin with a review of thermochemical conversion technologies with an emphasis on the feedstock properties that are important for each technology and relate these properties back to biomass traits that are commonly measured by biologists. This is followed by a discussion of the natural variation in plant traits that can be exploited for optimization of these properties, including what is known of the genetics governing those traits, and the potential impacts of modifying these traits at a systems level. We end with a discussion of how best to measure these properties and traits, and offer a perspective on which approaches might be useful for high-throughput phenotyping. To help relate the different biomass traits that biologists and engineers measure, we provide a brief list of terms and definitions (Table 1). Areas where there are large gaps in knowledge are highlighted as future research needs. Our focus is on cellulosic biomass from herbaceous crops because (1) herbaceous agricultural residues comprise a large potential resource (DOE, 2011), (2) a large fraction of the US biofuel mandate is expected to be dedicated herbaceous bioenergy crops (DOE, 2009; USDA, 2010), and (3) herbaceous crops can be grown in more regions than woody crops, and allow more flexibility in year to year land allocation.

**Table 1:** **Common terms used in this review in the context of biomass for bioenergy**.

Biochemical analysis	Characterization of biomass in terms of structural and non-structural carbohydrates, lignin, protein, and extractives (pectins, lipids, etc.)
Enzymatic conversion	Use of microorganisms or pure enzymes to transform feedstocks into energy products and co-products, e.g., fermentation, anaerobic digestion
Fixed carbon (FC)	Mass remaining as a solid after proximate analysis, excluding ash
Higher heating value (HHV)	Energy released as biomass undergoes complete combustion to CO_2_, H_2_O (condensed), and other minor products at standardized conditions
Intensive properties	Non-separable traits that are independent of the mass of a sample
Property	Trait or parameter in the context of a certain bioenergy conversion pathway or engineering systems
Phenotype	Observable or measurable characteristic specific to a given environment
Proximate analysis	Characterization in terms of the mass volatilized (as moisture and volatile matter) and mass remaining (fixed carbon and ash) during a standardized heating regime
Summative properties	Traits that describe specific separable components of the biomass and sum to 100% in the context of a mass balance
Thermochemical conversion	Controlled heating or oxidation of feedstocks to produce energy products and/or heat, e.g., pyrolysis, gasification, combustion
Trait	Genetic or physical characteristics (physical characteristics are also referred to as phenotypes)
Ultimate analysis	Characterization of biomass in terms of its individual constituent elements (C, H, O, N, S, etc.)
Volatile matter (VM)	Mass loss as gaseous products (excluding moisture) during proximate analysis

## Feedstock properties for thermochemical conversion

### Thermochemical conversion technologies

Thermochemical conversion is the controlled heating and/or oxidation of biomass as part of several pathways to produce intermediate energy carriers or heat (Figure 1). Included is everything from biomass combustion, one of the simplest and earliest examples of human energy use, to experimental technologies for the production of liquid transportation fuels and chemical feedstocks. Thermochemical conversion technologies are classified by their associated oxidation environment, particle size and heating rate, ranging from heating biomass in an oxygen-free environment (endothermic) to full exothermic oxidation of biomass.

Pyrolysis is the thermal decomposition of biomass into highly heterogeneous gaseous, liquid, and solid intermediates in the absence of oxygen; the process is endothermic. The liquid product (pyrolysis oil) is a heterogeneous mixture characterized by high oxygen content and alkalinity, which can be upgraded to fuels or chemicals. The solid product (char) can be used as a fuel or soil amendment (Field et al., 2013). Pyrolysis is differentiated between slow pyrolysis, with residence times ranging from minutes to days and optimized for the production of char whereas fast pyrolysis, with residence times on the order of seconds to minutes, is optimized for the production of pyrolysis oil (Babu, 2008). On the engineering front, research is focused on optimizing process variables (temperature, heating rate, oxidation environment) and product upgrading via catalytic and thermal processes to produce infrastructure-compatible liquid transportation fuels (Demirbas, 2007).

Gasification is the exothermic partial oxidation of biomass with process conditions optimized for high yields of gaseous products (syngas or producer gas) rich in CO, H_2_, CH_4_, and CO_2_. The gas can be cleaned and used directly as an engine fuel or upgraded to liquid fuels or chemical feedstocks through biological fermentation (Datar et al., 2004) or catalytic upgrading via the Fischer-Tropsch process (Boerrigter and Rauch, 2005; Huber et al., 2006; Wang et al., 2008). One of the challenges of gasification is the management of higher molecular weight volatiles that condense into tars; these tars are both a fouling challenge and a potential source of persistent environmental pollutants such as polycyclic aromatic hydrocarbons (Milne et al., 1998).

The direct combustion of biomass is still the dominant bioenergy pathway worldwide (Gaul, 2012). Complete combustion involves the production of heat as a result of the oxidation of carbon- and hydrogen-rich biomass to CO_2_ and H_2_O. However, the detailed chemical kinetics of the reactions that take place during biomass combustion are complex (Jenkins et al., 1998; Babu, 2008) and imperfect combustion results in the release of intermediates including environmental air pollutants such as CH_4_, CO, and particulate matter (PM). Additionally, fuel impurities, such as sulfur and nitrogen, are associated with emission of SO_X_ and NO_X_ (Robbins et al., 2012).

Other thermochemical technologies include carbonization, the production of charcoal via the partial oxidation of woody feedstocks with long residence time (Bailis, 2009), and hydrothermal approaches, which utilize an aqueous environment at moderate temperatures (200–600°C) and high pressures (5–40 MPa) to decompose biomass into solid, liquid, and gaseous intermediates (Peterson et al., 2008; Brown, 2011). Another technology, torrefaction, is the low temperature (200–300°C) pyrolysis of biomass in order to remove water and volatiles, increasing its energy density and susceptibility to mechanical pretreatment (Van der Stelt et al., 2011). The remainder of this review will focus on pyrolysis, gasification, and combustion, as these are the most fully developed modern bioenergy pathways with the most clearly defined feedstock requirements.

### Relationships between feedstock properties

**Figure 2:**
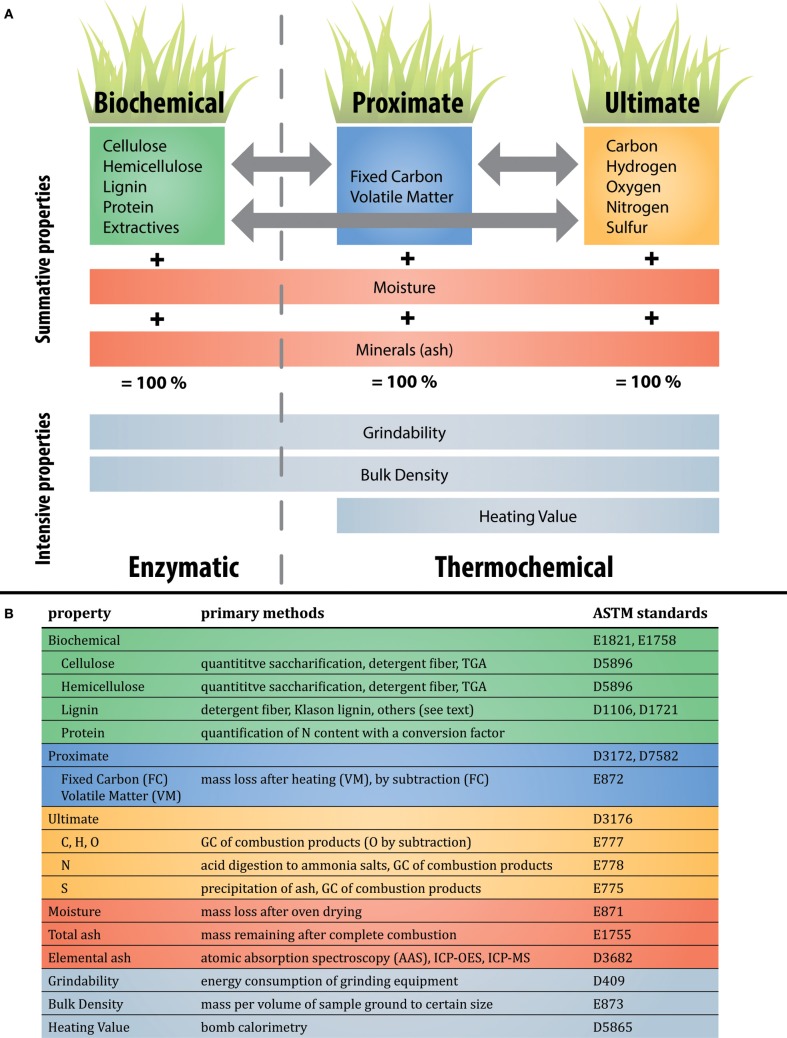
**Overview of the relationships between biomass traits and properties, and common methods of quantification**. Colors for each property are maintained throughout Figure 2. **(A)** Biomass characterization in terms of summative properties (shown in green, blue, orange, and red) and intensive properties (shown in gray). Three common paradigms for describing biomass are inter-related: biochemical, proximate, and ultimate. While enzymatic conversion has focused on characterizing biomass in a biochemical paradigm, two alternatives more appropriate for thermochemical conversion are proximate and ultimate analysis. Moisture and minerals (ash) are common across all paradigms. **(B)** Examples of common primary (direct) methods of quantifying each component identified in **(A)**. Note that this list is not complete, and note that proximate analysis necessitates moisture and total ash quantification. Elements that remain in the ash when biomass is combusted are referred to as minerals before combustion and ash afterwards. Examples of relevant ASTM standards for biomass, wood, refuse, or coal are listed. These direct methods are contrasted with indirect methods described in the text but not shown here.

The performance of these thermochemical conversion pathways relies on the use of appropriate biomass feedstocks. The mass balance of a kilogram of biomass is commonly conceptualized in three different ways, via either biochemical, proximate, or ultimate analysis (Figure 2A). Biochemical analysis refers to the relative abundance of various biopolymers (e.g., cellulose, lignin, etc) in the biomass, whereas ultimate analysis refers to the relative abundance of individual elements (e.g., C, H, O, N, and S). Proximate analysis involves the heating of biomass to quantify its thermal recalcitrance via the relative proportions of fixed carbon (FC) and volatile matter (VM), a method originally designed for the characterization of coal (e.g., American Society for Testing and Materials, ASTM standard D3172). These different conceptualizations are alternate ways to describe the same biomass; for example, a higher lignin:cellulose ratio (biochemical) also implies lower H:C and O:C ratios (ultimate) (Couhert et al., 2009). Moisture and elemental ash complete the mass balance of a unit of freshly-harvested biomass, and are universal across these different conceptualizations. Different combinations of these mass-based properties (summative properties) result in different bulk properties (intensive properties) such as grindability (comminution), density and heating value.

Feedstock properties that affect thermochemical conversion effectiveness include heating value, ash content, moisture level, and others discussed next. While thermochemical conversion engineers typically describe biomass in terms of proximate or ultimate analysis, biologists and breeders are more accustomed to the terminology of biochemical analysis. Thus, important properties are introduced in the context of proximate/ultimate analysis, and then related back to their biochemical equivalents. Current knowledge of the genetic and environmental control of these biochemical properties are then described in detail in the section titled “Genetic Control of Traits Related to Feedstock Properties.”

### Heating value and ratios of C, H, and O

Heating value, also known as calorific value, is the energy available in the feedstock as estimated from the heat released during complete combustion to CO_2_, H_2_O (gaseous H_2_O for lower heating value, LHV, or liquid H_2_O for higher heating value, HHV), and other minor products (N_2_, ash, etc.), and is a primary measure of quality of a feedstock. Moisture content impacts the useful energy of freshly harvested biomass as heat liberated during combustion is wasted evaporating this moisture (Bridgwater et al., 2002). Since HHV is a mass based measurement, high mineral content leads to a decrease in HHV, because minerals contribute little energy during biomass oxidation (Jenkins et al., 1996; Sheng and Azevedo, 2005). This is particularly important for grasses and other herbaceous feedstocks that can consist of up to 27% ash by mass (Table 2).

**Table 2:** Ranges of key thermochemical properties in several biomass feedstocks as summarized from literature^*^.

	**Corn stover^a^**	**Corn cob^b^**	**Wheat straw^c^**	**Rice straw^d^**	**Sugarcane bagasse^e^**	**Reed canarygrass^f^**	**Switchgrass^g^**	**Miscanthus^h^**	**Poplar^i^**
**BIOCHEMICAL**									
Cellulose (%)	28–51	26–36	25–51	28–41	32–43	26–39	30–50	41–58	39–49
Lignin (%)	11–21	6–17	8–30	10–23	19–28	4–14	5–23	8–22	18–32
**PROXIMATE**									
Fixed carbon (%)	15–20	17–19	15–22	15–25	12–20	16–24	13–27	5–26	12–28
Volatile matter (%)	72–85	80–83	71–85	64–98	74–88	73–83	73–87	74–94	72–86
Moisture (%)	11–33	12–55	8–15	3–74	16–50	15–25	40–70	20–52	8–59
Total ash (%)	4–10	1–9	1–23	8–26	1–13	3–13	2–10	1–9	0.4–4
**ULTIMATE**									
C (%)	40–51	41–50	42–53	35–60	38–55	44–50	42–53	40–52	47–52
H (%)	4.7–6.3	5–7.4	3.2–9.8	3.9–7	5.3–6.7	5.2–6.5	4.9–6.5	4.4–6.5	5.6–6.3
O (%)	34–50	44–51	29–52	31–50	33–50	39–49	36–49	39–49	40–46
O:C molar ratio	0.50–0.94	0.66–0.93	0.43–0.93	0.38–1.07	0.58–0.99	0.59–0.84	0.51–0.88	0.56–0.92	0.58–0.74
H:C molar ratio	1.10–1.91	1.21–1.95	0.73–2.83	0.79–2.42	1.23–2.13	1.26–1.79	1.12–1.87	1.02–1.97	1.30–1.62
**MINERAL (ASH) COMPOSITION**									
Al_2_O_3_ (% ash)	0.1–5	0.8–5	0.1–12	0.1–3.39	5–21	0.2–2	0.12–7	0.1–3	0.2–3
CaO (% ash)	5–15	0.5–15	3–17	0.7–10	2–19	0.5–10	5–14	3–14	29–61
Cl (% ash)	0.3–1.9	–	0–7.2	0.6^**^	0.03^**^	0.06^**^	0.1–0.6	0.03–7	0.01–0.03
Fe_2_O_3_ (% ash)	0.4–2.5	0.2–7	0.7–2.2	0.1–3	2–16	0.2–1.7	0.35–3.6	0.08–2.6	0.3–1.4
K_2_O (% ash)	15–21	2–20	6–37	6–25	0.15–20	2–23	5–28	2–34	10–34
MgO (% ash)	1.9–10	2.5–6	0.8–4	0.8–5.8	1.9–12	0.01–5	2.6–6.5	0.9–12	0.1–18
Na_2_O (% ash)	0.2–1.5	0.2–1.8	0.1–17	0.2–4	0.4–1.6	0.03–2.3	0.1–1.9	0.1–2.3	0.1–0.4
P_2_O_5_ (% ash)	1.9–9	0.7–10	1.2–8	0.7–9	0.9–3.2	0.4–14	2.6–15	1.5–29	0.9–8
SiO_2_ (% ash)	50–69	40–75	27–73	50–82	46–58	37–95	46–70	26–86	3–9
SO_3_ (% ash)	0.8–13	1.4–13	1.2–8	0.7–6	0.4–3.8	0.02–2.1	0.4–9	0.6–5	2–3.8
TiO_2_ (% ash)	0.2–0.3	–	0.01–.22	0.01–0.09	2.6–3.8	0.05–5	0.09–.37	0.02–0.05	0.3^**^
Alkali index (kg alkali oxide/GJ)^j^	–	–	1.1–1.7	1.4–1.6	0.06^**^	–	0.6^**^	–	0.14^**^
**OTHER PROPERTIES**									
Higher heating value (MJ/kg)	18–20	16–19	12–22	15–20	19–20	18–21	17–20	17–22	17–21
Bulk density (kg/m^3^)	66–131	195^**^	51–97	63–75	50–75	–	65–105	70–100	–

* Ranges are combinations of species and/or hybrids, and include different environments, soils, treatment conditions, contamination, experimental error, etc. Values <4 were rounded to 1 decimal place, values >4 were rounded to whole numbers (except for ratios, and values used to calculate ratios). O:C and H:C were calculated by taking the % C, H, and O, and dividing by the atomic masses for each element to give % molar mass, then dividing the min by the max to get the global min, and the max by the min to get the global max. Where possible, values reported are on a dry matter basis, and using similar methods. Comparing values across methods is especially problematic for bulk density, moisture, cellulose, and lignin as standardized methods are not always practiced or described, and some methods are more accurate than others.

** Only individual values were found in the literature review.

a Mani et al., 2004; Oak Ridge National Laboratory, 2008; Petrolia, 2008; Carpenter et al., 2010; Chevanan et al., 2010; Vassilev et al., 2010; Energy Research Centre of the Netherlands, 2012; Tao et al., 2012a,b; Zhao et al., 2012a.

b Smith et al., 1985; Coovattanachai, 1989; Spokas, 2010; Energy Research Centre of the Netherlands, 2012; Tao et al., 2012a,b; Zhao et al., 2012a.

c Jenkins et al., 1998; McKendry, 2002a; Mani et al., 2004; Lam et al., 2008; Carroll and Somerville, 2009; Wu et al., 2009; Carpenter et al., 2010; Chevanan et al., 2010; Spokas, 2010; Energy Research Centre of the Netherlands, 2012; Tao et al., 2012a,b.

d Jenkins et al., 1998; Wu et al., 2009; Allison et al., 2010; Kargbo et al., 2010; Vassilev et al., 2010; Jahn et al., 2011; Liu et al., 2011; Energy Research Centre of the Netherlands, 2012; Tao et al., 2012a,b; Zhang et al., 2012; Zhao et al., 2012a.

e Jenkins et al., 1998; Kaar et al., 1998; Tsai et al., 2006; Spokas, 2010; Vassilev et al., 2010; Energy Research Centre of the Netherlands, 2012; Tao et al., 2012a,b.

f Fahmi et al., 2008; Lindh et al., 2009; Allison et al., 2010; Vassilev et al., 2010; Energy Research Centre of the Netherlands, 2012; Tao et al., 2012a,b.

g Jenkins et al., 1998; McKendry, 2002a; Mani et al., 2004; Lam et al., 2008; Sokhansanj et al., 2009; Carpenter et al., 2010; Chevanan et al., 2010; Tao et al., 2012a.

h Clifton-Brown and Lewandowski, 2002; Allison et al., 2010; Arabhosseini et al., 2010; Energy Research Centre of the Netherlands, 2012; Tao et al., 2012a,b.

i Jenkins et al., 1998; Klasnja et al., 2002; Carroll and Somerville, 2009; Spinelli et al., 2009; Vassilev et al., 2010; Tao et al., 2012a; Energy Research Centre of the Netherlands, 2012; Tao et al., 2012b; Zhao et al., 2012a.

j Alkali index is a ratio calculated from the relative amounts of K_2_O and Na_2_O. See text or Jenkins et al. (1998) for detailed explanation.

Biomass feedstocks are also described in terms of ultimate analysis based on the relative content of individual elements such as C, H, and O. The overall ratios of these elements are directly related to the biochemical components of the cell wall. Cellulose has a higher H:C and O:C ratio than lignin (Couhert et al., 2009). Lignin has a higher HHV than cellulose or starch (Helsel and Wedin, 1981; Demirbaş, 2001), consistent with the idea that oxygenated fuels release less heat on combustion (Vermerris and Saballos, 2013). This is an example of divergent feedstock requirements for enzymatic vs. thermochemical conversion pathways: while minimizing lignin improves hydrolysis and fermentation yields, high lignin is beneficial for the energy balance of thermochemical systems.

Upgrading gaseous pyrolysis and gasification products to liquid fuels also requires a specific H:C stoichiometry (Datar et al., 2004; Wright and Brown, 2007). Biomass has a low H:C ratio (ranging from 0.7 to 2.8 in Table 2) relative to that of the desired liquid products (2–4 for alcohols and alkanes), so full conversion requires adding supplemental hydrogen in the form of steam or H_2_, or removing carbon as CO_2_ (Borgwardt, 1999; Pereira et al., 2012). High lignin levels may be advantageous for thermochemical conversion pathways targeting liquid fuels, as it may move the process closer to overall stoichiometric balance.

### Proximate analysis and conversion product yields

Proximate analysis separates the biomass into four categories of importance to thermal conversion: moisture, VM (gases and vapors driven off during pyrolysis), FC (non-volatile carbon), and ash (inorganic residue remaining after combustion) (Miles et al., 1996; Jenkins et al., 1998; Riley, 2007). The measurement is a proxy for thermochemical conversion performance, and the relative proportions of FC vs. VM are related to the relative yields and composition of solid, liquid, and gaseous products generated during pyrolysis and gasification (Brar et al., 2012). Even for combustion, the FC:VM ratio may significantly change the emissions profile of products of incomplete combustion (Cummer and Brown, 2002). Biomass generally contains high levels of VM (ranging from 64 to 98%, Table 2) compared to fossil coal [typically below 40% (Vassilev et al., 2010)].

In addition to impact on heating value, the relative concentrations of cellulose and lignin also affect the yields of thermochemical conversion products. The different biochemical constituents of biomass have different levels of thermal stability, and as pyrolysis temperatures increase hemicellulose reacts first, followed by cellulose and then lignin (Fahmi et al., 2007; Gani and Naruse, 2007). This is consistent with studies that show isolated lignin extracts having a higher FC content than pure cellulose (Couhert et al., 2009), a strong positive correlation between FC and lignin across multiple biomass samples (Demirbaş, 2003a), and increasing lignin levels associated with low gas yields and high char yields during fast pyrolysis (Lv et al., 2010). However, several studies suggest the opposite, showing cases where increasing lignin is associated with lower FC (Fahmi et al., 2007, 2008), or increasing yields of pyrolysis oils (Tröger et al., 2013).

Clear relationships between FC:VM and lignin:cellulose content in biomass samples are likely confounded by the presence of minerals, some of which exert a strong influence on the yields and qualities of thermochemical conversion products due to catalytic activity (Couhert et al., 2009; Lv et al., 2010). For pyrolysis, high mineral content reduces oil yield and increases char and gas products (Fahmi et al., 2007; Couhert et al., 2009; Tröger et al., 2013). Relationships between VM and lignin are confounded by ash content (Raveendran et al., 1995). In addition, ash exerts a catalytic effect on the liquid fraction, encouraging cracking of high molecular weight species into lighter ones (Fahmi et al., 2008). The catalytic activity of ash changes the dynamics of combustion and gasification; reducing the ash content of biomass by washing has been shown to increase the temperature of peak combustion rate (Fahmi et al., 2007) but decrease the temperature of peak gasification mass loss rate (Lv et al., 2010). Many studies show a negative correlation between mineral content and lignin across many types of biomass (Fahmi et al., 2007, 2008; Lv et al., 2010). Thus, the relationship between ash, lignin, and pyrolysis product yield is complex and careful experimental manipulation will be necessary to determine the causality underlying the observed correlations of low ash, high lignin, and high yields of heavy liquid products (Fahmi et al., 2008; Couhert et al., 2009).

### Other effects of mineral content

Besides lowering the heating value of biomass and changing the distribution of conversion products, mineral and elemental ions that plants accumulate can interfere with the operation of thermochemical conversion equipment. The elements in plant biomass volatilize during combustion and form a liquid slag or solid deposits as they cool (Miles et al., 1996). The elements Na, K, Mg, Ca as well as Cl, S and Si are the most problematic for thermochemical processes (Miles et al., 1996), and the combination of alkali metals with silica can form alkali silicates (McKendry, 2002b)—see Box 1 for more information regarding silica. The Cl in biomass can also be a significant problem because it interacts with vaporized metals, shuttling them to boiler surfaces where they form sulfates (Allison et al., 2010). Cl can also lead to elevated HCl and dioxin emissions (Lewandowski and Kicherer, 1997). As volatile gases combine, they form corrosive deposits that degrade components of the boiler. Other interactions can occur between the elements in biomass and coal when co-fired (Dayton et al., 1999). Since gasification can occur at lower temperatures, the severity of these issues might be reduced with that process; however, other issues can become more severe [Mansaray et al., 1999 and see Lv et al. (2010) for discussion]. Although difficult to generalize due to the complex and unique interactions that occur in each feedstock, ash content above 5% is probably unacceptable (McKendry, 2002c) and element specific recommendations are listed elsewhere (Van Loo and Koppejan, 2008). The alkali index (kg K_2_O and Na_2_O per GJ energy) can be used to predict performance in a thermochemical setting (Jenkins et al., 1998). With an alkali index above 0.17 kg/GJ, fouling is probable, and above 0.34 kg/GJ, it is almost certain. Several other indices exist, but were created for coal, so may not be good predictors for biomass (Yin et al., 2008). High feedstock mineral content can be mitigated to a certain extent by using newer alloys to construct components that can minimize and withstand some corrosion, and controlling the temperature of the reaction (Jenkins et al., 1998; Fahmi et al., 2008).

Box 1 **Silica in Grasses: Example and Opportunity.**

We present silica here as a practical matter—in grasses it can represent a large proportion of ash content—and as an example of how existing genetic knowledge might be leveraged to optimize a thermochemical trait in feedstocks. Silica does not provide energy during thermochemical conversion, hence it lowers the energy density. Furthermore, silica reacts with other alkali metals such as potassium and forms alkali silicates that have a lower melting point, thereby increasing the slagging and deposition rates at lower temperatures (Wang et al., 2008). Manageable silica levels are difficult to estimate, since it depends on the levels of other alkali metals in the biomass. However, for many grasses, lowering silica levels at least below the 5% ash threshold would improve the thermochemical potential of these grasses.

Some have argued to include silicon as an “essential” element (Epstein, 1999) due to its important and diverse roles. Silica serves as a structural element, keeping leaves erect and stems from lodging. Its physiological roles include detoxifying Al, Mn, and Fe by binding with them and regulating P uptake (Richmond and Sussman, 2003), decreasing transpiration and reducing water stress (Ahmad et al., 1992; Epstein, 1999) and in its protective role, it may provide a mechanical barrier that hinders diseases and pests (Winslow et al., 1997; Richmond and Sussman, 2003; Cotterill et al., 2007; Keeping et al., 2009). These roles of silica have been validated in many diverse species such as rice, sugarcane, barley, jute, tomato, cucumber and strawberry (Datnoff et al., 2001).

Although the second most abundant element in the world's soils, silicon is not always in a form available to plants (Sommer et al., 2006). Soil water concentrations of monosilicic acid (H_4_SiO_4_), the plant available form of silica, vary from 0.1 to 0.6 mM in most soils (Datnoff et al., 2001). Silica deficiency is rare, but in sandy and highly weathered soils, and intensely cultivated soils, silica application can improve yields (Datnoff et al., 2001; Ma and Takahashi, 2002). In most plants, silica, an uncharged molecule in biological conditions, is taken up with the water stream and diffuses through membranes, following the transpiration stream up the xylem (Mitani et al., 2005). It is deposited as “opal” or “phytoliths,” more accurately called amorphous silica (SiO_2_ · nH_2_O), usually where transpiration has caused the solution to become saturated—in the intercellular spaces and the bulliform cells. Deposition also occurs frequently in silica bodies, xylem cells, root endodermis cells, and in the cuticle silica double layer along the epidermis of leaf blades (Yoshida et al., 1962; Prychid et al., 2003). It is becoming clear that silica deposition can be a carefully engineered process directed by the plant, as temporal control of silica deposition in silica bodies demonstrates (Zhang et al., 2013).

Silica content of plants ranges from trace (less than 0.5%) to small (0.5–1%, roughly corresponding to the amounts in the soil water), to high (1–15%) amounts (Raven, 1983; Datnoff et al., 2001; Ma and Takahashi, 2002). Accumulation of high levels seems to require an active system of transporters. For example, rice accumulates high levels of silica via characterized transporters, including an aquaporin in root cells, an antiporter that uses the proton gradient to load silica into the xylem, and a passive transporter that moves silica from the xylem to the leaf (Ma et al., 2011). Several bioenergy feedstocks accumulate high silica levels (see Table 2) but the specific transporters are yet to be identified and the effects of modifying their production or activity are unknown. It is important to note for practical purposes additional silica may be introduced into the feedstock with soil contamination of the biomass.

While there is usually a correlation between soil-available silica and amounts of silica taken up by plants, there is large variation for the amount accumulated, even within a species. When grown the same soil, some varieties of rice always accumulate more silica than other varieties (Deren et al., 1992). In general, japonica rice varieties take up more silica than indica varieties, maybe because the japonica types were domesticated on silica deficient soils (Datnoff et al., 2001). Plants that are non-accumulators (corresponding to the trace levels discussed above) do not take up silica—even under high soil silica conditions. It is unclear why plants have adapted to maintain such different levels of silica. Cell specific deposition indicates that silica is under genetic control (Yoshida et al., 1962; Duan et al., 2005; Monti et al., 2008). Quantitative trait loci (QTL) have been mapped for silica concentration in various tissues (Wu et al., 2006; Dai et al., 2008) and there are hints that some disease resistance genes may actively modify silica levels (Li et al., 2012) and different types of silica deposition may have different roles (Isa et al., 2010).

Silica content is estimated in plant tissue by hydrofluoric acid extraction and a molybdenum blue assay (Saito et al., 2005) or by gravimetric techniques (Datnoff et al., 2001). Measurement is also possible with ICP-OES (El-Nashaar et al., 2009) and distribution within a tissue can be assessed by X-ray fluorescence spectroscopy (Datnoff et al., 2001). In all cases, care must be taken to avoid glassware that could introduce additional Si into the sample. Since the large majority of ash in many grasses is silica, crude measures of ash analysis can correlate with silica content. Additionally, ash can be predicted via NIR spectroscopy and these indirect methods (crude ash and NIR) might be optimized for high-throughput measurement of silica.

In conclusion, the observations of natural variation in silica content and the discovery of specific targets, the silica transporters, indicate the potential of silica levels as a target for biomass crop improvement. Indeed, in a study of ash levels across 144 species, Tao et al. identified silica content as a good target for optimized biomass (Tao et al., 2012a). A targeted approach might be to simultaneously decrease silica content: possibly by downregulating silica transporters, while upregulating lignin production to compensate for the loss of silica. However, it will be important to monitor plant performance as silica levels are manipulated because silica can be critically important for plant growth and yield.

### Moisture content

Moisture content is a measure of the amount of water in biomass and is usually expressed as percent mass (wet basis). In addition to reducing the net heating value as discussed previously, high moisture content can reduce the effectiveness of individual thermochemical conversion processes. For combustion or co-firing, low moisture content, preferably around 5%, is desired because incomplete combustion can occur when the moisture content is too high. Some systems such as fluidized bed combustors are more flexible, and allow up to 35% moisture (Bridgwater et al., 2002). For gasification, acceptable moisture content can be as high as 20% or 30% (Cummer and Brown, 2002), but more commonly is around 15% moisture. For pyrolysis, initial moisture content contributes to the water content in the pyrolysis oil and above around 10% moisture, the oil produced will separate into two phases (Brar et al., 2012; Solantausta et al., 2012). For hydrothermal conversion, wet biomass can be used without drying, but these technologies are still in the development stages (Yoshida et al., 2004; Waldner and Vogel, 2005; Peterson et al., 2008; Pereira et al., 2012).

### Other considerations

In general, biomass has low amounts of S relative to fossil fuels, which minimizes SO_X_ pollution from gasification or combustion systems and avoids catalyst poisoning in fast pyrolysis systems (Brown, 2011). It can have similar or higher N, which contributes to NO_X_ emissions, but this can be mitigated to some extent through engineering in the process, e.g., by the use of exhaust scrubbers (Yin et al., 2008). High levels of nitrogen can also be problematic for the quality of liquid fuel products from fast pyrolysis (Wilson et al., 2013). For combustion processes, lignin is associated with PM emissions (Williams et al., 2012), a factor that must be balanced against the associated increase in feedstock HHV from a systems perspective.

In addition to direct effects on thermochemical conversion performance, biomass properties are also relevant to the upstream logistics associated with biomass transport and mechanical pre-treatment. Minimizing moisture reduces weight during transport from the field, and maximizing dry bulk density allows more cost effective transport of biomass. It has been estimated that reducing moisture content from 45 to 35% in biomass can lead to a 25% increase in the net present value of a thermochemical project producing ethanol from cellulosic biomass—mostly by reducing the energy and cost of drying the biomass (Gonzalez et al., 2012). Grindability relates to many other properties including moisture content and composition (Ghorbani et al., 2010). Beyond impacts on biomass transport costs, bulk density can influence how easily biomass can be ground for processing (Cabiles et al., 2008).

## Genetic control of traits related to feedstock properties

As highlighted in Figure 2A and introduced in the previous section, feedstock properties are related to biochemical traits that have been the focus of research by the forage, pulp and paper industry, as well as enzymatic bioenergy research for many years. These biochemical traits are more easily explained in the context of genes that encode the proteins that synthesize and deliver the components of the cell wall as well as the enzymes responsible for assembly of the wall components into complex structures.

For breeding or biotechnology approaches to improve cell wall composition, a major constraint is understanding which genes or gene pathways are important. Relating genotype to phenotype, i.e., to assign a gene responsible for a particular phenotype, allows identification, functional analysis, and modification of the gene (or its regulation) to improve the phenotype. For example, experiments that modify genes individually and in combination show the effect of a given gene on the composition of the biomass (Jung et al., 2012; Wang and Dixon, 2012; Yang et al., 2012). This information can be the basis for development of molecular markers to improve the phenotype by breeding or to design gene constructs for improvement through biotechnology. This knowledge, frequently gained from model plants can be applied even to distantly related species by using comparative genomics approaches (Ficklin and Feltus, 2011). This is important because for some species, notably several emerging energy grasses, genetic tools are just being developed. As with all breeding efforts, agronomic considerations must be considered; that is, the plants must still be able to survive and produce an acceptable yield. In the following sections, we discuss the genetic and environmental control of traits related to thermochemical conversion properties.

### Cellulose and lignin

Often comprising more than 50% of the cell wall, cellulose and lignin have been well-studied and the enzymes involved in their synthesis are well understood (Boerjan et al., 2003; Endler and Persson, 2011). However, how these components are linked within the cell wall, and how the synthesis and modification are regulated are not well understood (Zhao and Dixon, 2011). There is a complex balance between cellulose and lignin levels, and the manipulation of genes involved in their biosynthesis sometimes leads to unexpected results (Gallego-Giraldo et al., 2011). Plants are surprisingly flexible, and can utilize a diverse set of precursors to build their cell walls. For example, Jensen and coworkers modified the native form of xyloglucan (a hemicellulose) in *Arabidopsis* without any apparent phenotypic consequences (Jensen et al., 2012). Yang and colleagues engineered plants to have thicker cell walls with more polysaccharides, but less lignin without negative consequences (Yang et al., 2012).

Research has focused on genes controlling the wall composition of the model dicot, *Arabidopsis*, or woody crops like poplar. However, to apply knowledge of these genes to more feedstocks, the findings will need to be validated in new crops. For example, lignin monomer composition differs between woody and herbaceous crops (Grabber et al., 2004; Buranov and Mazza, 2008). Gymnosperms have mostly G lignin while dicots have G and S and monocots generally have all three types. These monomers have different properties, including different estimated HHV (Amthor, 2003), and may influence the thermochemical properties of the biomass (Shen et al., 2010). It has been found that coniferous (mostly G) lignin is more thermally-stable than deciduous (mostly S) lignin (Müller-Hagedorn et al., 2003), and this is likely because G lignins contain more resistant linkages than S lignins (Boerjan et al., 2003). Approaches to fine-tune lignin composition have been suggested (Weng et al., 2008). The ratio of these monomers, as well as the soluble phenolics, may have consequences as important as cellulose and lignin ratios (Gani and Naruse, 2007; Shen et al., 2009; Studer et al., 2011; Elumalai et al., 2012). Because lignin biosynthesis genes vary across plant families, and between dicots and monocots, (Xu et al., 2009), it is likely that other unexamined differences in lignin composition in crop species might exist (Xu et al., 2009). In addition to the three major lignin monomers, monocots contain relatively large amounts of soluble phenolics and the genes controlling these might be useful targets to modify cell wall composition (Ishii, 1997; Bartley et al., 2013; Molinari et al., 2013).

Beyond genetically controlled variation of wall composition within and between species, growth environment plays a large role. Adler and colleagues observed that lignin content increased from 10 to 33% between a fall and spring harvest of the same crop of switchgrass (Adler et al., 2006). Monono and colleagues observed differences in total yield, composition, and ethanol yield in switchgrass between locations and seasons (Monono et al., 2013). Miscanthus also displays variation in composition across environments (Allison et al., 2011). Switchgrass S, G, and H monomer ratios show major differences when grown in the growth chamber, greenhouse or field (Mann et al., 2009), which is consistent with strong genotype by environment interactions (Hopkins et al., 1995; Lemus et al., 2002). Sugarcane internode composition changes over the growing season (Lingle and Thomson, 2011). Thus, although a viable focus, optimization of biomass through manipulation of wall lignin and cellulose composition and content will require not only an understanding of the genetic controls for these components, but also significant knowledge of the environmental component.

### Mineral content and elemental ash

Elements commonly found in biomass ash are profiled in Table 2. There are major differences in the concentrations of these elements between woody and herbaceous crops, and herbaceous crops generally have more N, Cl, and K, but less Ca than woody crops (Vassilev et al., 2010; Tao et al., 2012a). Though not essential for survival, Si is accumulated to high levels in many grasses, up to 10% dry weight (Epstein, 1999). Vassilev and colleagues find that levels of elements seem to exist in five associated groups in biomass, and these associations may have underlying biological significance: C–H; N–S–Cl; Si–Al–Fe–Na–Ti; Ca–Mg–Mn; and K–P–S–Cl (Vassilev et al., 2010). Therefore, attempting to modulate Ca levels for example, might also impact Mg and Mn levels and it might be difficult to breed away from these associations. In addition to individual elemental associations, there is also evidence of a relationship between total ash content and biochemical constituents, with total ash content inversely proportional to lignin (Fahmi et al., 2007, 2008), and total ash proportional to cellulose (Lv et al., 2010). It has been hypothesized that this relationship is due to overlap in the roles of lignin and mineral fraction with regard to mechanical stability and resistance to attack (Fahmi et al., 2007).

While the uptake, transport and roles of several of these mineral elements in plants are well understood (Taiz and Zeiger, 2006), little is known about the genes controlling variation for these traits (Mäser et al., 2001; Raboy, 2003; Ghandilyan et al., 2006). Uptake and distribution of these elements through the plant occurs via many different pathways, including uptake from the rhizosphere, transfers from roots to shoots, and remobilization among organs. These transport pathways can be both shared and opposing between elements, as indicated by positive and negative correlation of mineral and micronutrient phenotypes [reviewed by Ghandilyan et al. (2009)]. For example, Si is negatively correlated with Ca in some species (Nishimura et al., 1989), and reducing Si may simply increase Ca in plant tissues (and the Ca associated thermochemical issues). Cl content varies between stems and leaves of miscanthus (Lewandowski and Kicherer, 1997), and Cl and Ca variation has been observed in the bark, needles, and wood of various tree species (Werkelin et al., 2005). Tissue specific differences in other elements probably exist—indicating genetic control. Heritability for mineral content ranges from 10 to 90%, so breeding for some elements will be more difficult than others (Ghandilyan et al., 2009). Understanding variation for these traits among cultivars of switchgrass is complicated by strong environmental interactions (Lemus et al., 2002; El-Nashaar et al., 2009), as is probably the case for other feedstocks.

Elemental concentrations also vary widely between and within species, by tissue type, and across harvest time and environments (Landström et al., 1996; Adler et al., 2006; Boateng et al., 2006; Christian et al., 2006; Nassi o Di Nasso et al., 2010; Baxter et al., 2012; Singh et al., 2012; Zhao et al., 2012b). Of considerable importance when focusing on crop improvement in elemental composition is that any attempt at improvement will be complicated by the interaction of these gene pathways with other traits essential for crop productivity, i.e., agronomic traits such as drought and salt tolerance, disease or pest resistance (Egilla et al., 2001; Dordas, 2008; Baxter et al., 2009, 2010). Because the genetics is complex and the potential implications on agronomic traits are serious, focus has been on reducing the impacts of these elements by other solutions, such as adjusting harvest time (Monti et al., 2008), allowing the minerals to leach out in the field before collection (Jenkins et al., 1996), and adding compounds to minimize reactions during thermochemical conversion (Van Loo and Koppejan, 2008).

### Moisture content

Wet biomass from the field can contain greater than 50% moisture on a wet basis, but this can vary greatly (Table 2), and intrinsic moisture (water tightly bound to biomass) is much lower. Although moisture content is an important component of the energy content, the literature on genetic variation and alteration of traits governing moisture content are sparse. In several species of willow, differences in moisture content of up to 16% exist and almost 40% of this variation is due to genotype (Mosseler et al., 1988). In rice, moisture content between 20 diverse varieties varied from 43 to 74% and broad sense heritability was found to be 0.6 (Jahn et al., 2011).

It is well known that species and varieties of plants vary in their ability to cope with drought stress (Zhu, 2002; Golldack et al., 2011). One strategy that plants employ is to manipulate the osmotic potential of their cells, and thus allow water to be maintained under drought conditions (McCann and Huang, 2008). It is through this mechanism that genetic control of the moisture content of the cells exists, and thus possibly the plant as a whole at harvest time. Many of the genes involved in these processes have been characterized (Bartels and Sunkar, 2005). There may also be significant correlations between moisture content and mineral content, since minerals ions are utilized to modulate the osmotic potential of the cells (Patakas et al., 2002; Arjenaki et al., 2012). In rice varieties studied by Jahn et al. (2011), a correlation between leaf ash but not stem ash and moisture content was observed, although these relationships have yet to be directly examined.

Clearly there is evidence that selection for moisture content is feasible but application of genetic approaches to improving biomass crops for moisture content has remained largely unexplored. As for mineral content, agronomic solutions to minimizing moisture content have been employed. For example, post-senescence drying reduced moisture content by 30% in miscanthus stems (Hodgson et al., 2010).

### Other important traits

Other traits highlighted in Figure 2A but not discussed thus far in this section include HHV, grindability, bulk density, as well as components of proximate and ultimate analysis. While some information exists about their relationship with biomass composition, little information exists about the genetic control of these traits. Bulk density may be influenced by cell wall changes (Wang et al., 2010) and variation in grindability has been observed among corn stover, straw, and hardwood (Cadoche and López, 1989). The first steps toward studying these might be to measure their variation across a species (a genome wide association mapping study, GWAS), or study their segregation in a genetic mapping population (a QTL study) (Mackay, 2001; Collard et al., 2005; Takeda and Matsuoka, 2008; Zhu et al., 2008). A critical component of both of these approaches is the ability to measure these traits in large numbers of plants in a high-throughput manner.

## Potential for high-throughput phenotyping

We have identified many of the feedstock traits important for thermochemical conversion and discussed the relationships between traits. In this section, we review how these traits are measured, and in cases where several methods exist, we highlight those methods which might be amenable to high-throughput phenotyping of many individual plants.

### Biochemical analysis

The most complete approach to quantifying the cell wall content is quantitative saccharification (also referred to as dietary fiber, Uppsala method, or NREL method). Water and ethanol soluble fractions are isolated, followed by hydrolysis and quantification of the component sugars, sugar degradation products, and organic acids by high performance liquid chromatography (HPLC) or gas chromatography mass spectroscopy (GC/MS) and acid soluble lignin with UV-vis spectroscopy. Starch is quantified and subtracted from cellulose, since it would contribute glucose monomers and inflate the cellulose component. Protein, ash and acid insoluble lignin (Klason lignin) are quantified from the remaining residue (Theander et al., 1995; Sluiter et al., 2010). Another common method originally developed to determine forage quality is called detergent fiber or the Van Soest method, and involves treating biomass with various concentrations of acids and bases to sequentially hydrolyze components of the cell wall, followed by acid insoluble lignin and ash determination (Van Soest et al., 1991; Uden et al., 2005). Each method highlighted here assumes the monomeric sugars are derived from certain polymers in the cell wall, and each method has its own set of biases (Moxley and Zhang, 2007; Wolfrum et al., 2009).

While any method is probably feasible for high-throughput given enough investment in lab time, equipment or automation (such as robotics), we highlight recent approaches in lignin quantification and monomer composition with pyrolysis molecular beam mass spectroscopy (pyMBMS) (Mann et al., 2009; Studer et al., 2011) or thioglycolic acid lignin (Suzuki et al., 2009). Cellulose, hemicellulose and lignin have been estimated with a thermogravimetric analyzer (TGA) which is essentially a microbalance inside a controlled-atmosphere furnace (Serapiglia et al., 2009). High-throughput glycome profiling of cell wall extracts detects presence or absence of specific polysaccharides but does not quantify the various components (Pattathil et al., 2012). Pretreatment and saccharification approaches (Gomez et al., 2010; Santoro et al., 2010) or ethanol yield (Lee et al., 2012) directly test how amenable biomass is to enzymatic conversion, and indirectly provide information about the cell wall composition.

### Proximate analysis

Proximate analysis separates the biomass into moisture, VM, FC, and ash. This is accomplished through controlled heating of a ground sample in a furnace and observing mass lost during heating. VM and FC are determined after correcting for moisture and ash content. Proximate analysis can also be conducted in a single operation using a TGA. Heating value is also typically measured in the course of proximate analysis using bomb calorimetry, in which a biomass sample is fully combusted in a pure oxygen environment within a reaction vessel suspended in a water jacket; calorific value of the fuel is inferred from changes in the water temperature. HHV includes the energy released when the H_2_O produced during the combustion process condenses. An adjustment can be made since the energy due to water condensing is not captured in some systems—the adjusted value is the LHV.

While moisture content is part of standard proximate analysis procedure, it can also be evaluated by itself. The simplest, yet most time consuming method to assess moisture content is the oven dry method—moisture is removed by drying and the difference in mass is assumed to be moisture loss. These methods assume that the sample has been stored in an airtight container; otherwise moisture gain or loss (due to varying relative humidity of the storage environment) will have occurred between sample collection, and moisture determination. Often “as received” moisture is referred to in the literature—this is a meaningless value as it depends on the conditions that the sample underwent between the field and the lab and varies with humidity in the environment, and how long the plant was allowed to senesce in the field. Moisture content can also be estimated on a wet basis with handheld moisture meters (Jensen et al., 2006). These meters work by testing the conductance or capacitance of the material or various chemical means but only work in certain ranges of moisture (Bala, 1997). Biomass moisture is conceptually simple to understand and measure, but often goes unmeasured or unclearly reported, hampering our knowledge of the underlying genetic and environmental control.

### Ultimate analysis

Profiling the individual elements is accomplished with approaches that measure electronic properties of elements (absorption, emission, and fluorescence spectroscopy) or techniques that measure nuclear properties (radioactivity, mass spectroscopy). Elemental analyzers available from many manufacturers either flash oxidize or pyrolyze the biomass and measure products such as CO_2_, H_2_O, NO_x_ in the exhaust gas via gas chromatography and thermal conductivity in order to stoichiometrically back-calculate the initial concentrations in the biomass (see standards in Figure 2B). Profiling elements in the ash fraction has traditionally been accomplished by solubilizing the ash and detection with atomic absorption spectroscopy (AAS). This involves ionizing atoms using a flame and measuring the portion of light absorbed by the elements as they pass through the detector (Dean et al., 1997; Hoenig et al., 1998). When coupled with autosamplers, these instruments can be relatively high-throughput.

Recently, profiling the inorganic fraction in whole biomass (ionomics), has improved with advances in Inductively Coupled Plasma (ICP) techniques. These techniques ionize atoms in a plasma gas and measure emissions using Optical Emission Spectroscopy as the atoms fall to their ground state (ICP-OES), or the ionized atoms are passed to a mass spectrometer (ICP-MS) (Salt et al., 2008). ICP-OES can also be called ICP-AES (Atomic Emission Spectroscopy). Advantages with these approaches include sensitivity, small sample size, and the ability to quantify many elements from the same sample but quantification of some elements (notably Si; see Box 1 for further discussion) require special equipment and additional sample preparation.

### Other traits

Grindability is measured by recording the energy consumption of the equipment used to grind a sample to specified size (Mani et al., 2004; Abdullah and Wu, 2009). A standard procedure does not appear to exist but would be essential to develop before larger studies are undertaken because the trait is influenced by many factors including moisture content, particle size, and how tightly the biomass is packed before measurement (Lam et al., 2008; Chevanan et al., 2010). It should be possible to adapt the existing standard for testing and comparing different types of grinding equipment (ASTM E959) to compare different types of biomass using a standardized piece of equipment. Standard procedures exist for bulk density (Figure 2B), but are highly dependent on the initial particle size. Particle density, which excludes the air space between particles, is another technique to estimate density of biomass. This can be measured with a gas pycnometer that displaces the air between biomass particles with a known volume of gas (Sokhansanj et al., 2008).

### High-throughput phenotyping: automation and indirect measurements

Phenotyping biomass to distinguish between genetic and environmental controls on individual bioenergy traits requires the characterization of large populations of plants, and some of the techniques described above are more appropriate for analyzing large sets of samples than others. Detergent fiber analysis has been somewhat automated with filter bag systems (Vogel et al., 1999). Robotic systems that can grind and weigh many samples at once exist to determine properties important for enzymatic conversion (Santoro et al., 2010). Traditionally, protein is quantified with dyes (Bradford, Lowry, etc), with UV-vis spectroscopy, or other techniques reviewed in Sapan et al. (1999) or Noble and Bailey (2009), but indirect methods that quantify N (such as the Kjeldahl method or elemental analyzers) simply use a conversion factor to estimate crude protein (Mosse, 1990). There are a number of automated proximate analyzers, elemental analyzers, and calorimeters available (e.g., Fu et al., 2011; Kumar et al., 2011; Smets et al., 2011), in which multiple samples can be loaded into racks and then analyzed automatically by the instrument.

Another approach to high-throughput phenotyping is the identification of correlations between the trait of interest and others traits that are more easily measured. For example, heating value can be estimated based on biochemical, proximate, or ultimate analysis through various equations, summarized in Sheng and Azevedo (2005). Interestingly, ultimate analysis is the most reliable approach—maybe in part due to variation in estimating biochemical or proximate properties. It should be highlighted that like many regression approaches, the sample set that is used to build the equation is critical and thus the equations may be plant species specific. Since grindability is ultimately a function of properties like moisture and composition, equations can be used to predict it in various types of biomass (Ghorbani et al., 2010; Miao et al., 2011).

A variety of properties can also be predicted from non-destructive high-throughput spectroscopic methods, particularly infrared (IR), often measured with an instrument capable of utilizing a Fourier Transform approach (FTIR), or raman spectroscopy which provides information complementary with FTIR, and Near Infrared (NIR) methods. IR spectroscopy measures the absorption of IR radiation by functional groups within compounds and may be used to directly fingerprint the compound, or in complex samples (such as biomass) a predictive model can be developed to quantify the biomass composition. NIR spectroscopy provides information through the combinations of fundamental bond vibrations (harmonics and overtones) in many compounds that absorb different wavelengths of NIR radiation depending on their resonance structure and penetrates deeper into the sample than IR (Reich, 2005). Because of the complex interactions in the NIR spectra, it is generally necessary to develop a predictive model to correlate spectra with a primary analytical method to predict composition and may not be as sensitive as IR methods. Spectra and primary analytical quantification of the trait of interest is collected on a diverse set of representative samples and this is used to derive a calibration equation using multivariate statistical methods such as partial least squares (PLS) or principal component analysis (PCA) to correlate the spectra with the primary analytical methods. An excellent example of the range of assays that can be utilized as analytical methods to build NIR models is presented by Vogel et al., 2010. The equation is tested on another subset of samples to ensure that it accurately predicts the trait of interest basely solely on the spectra obtained (Sanderson et al., 1996; Burns and Ciurczak, 2001).

While there is a large initial investment in developing a model, the ability to predict composition of new samples based only on quickly capturing spectral information makes these methods an attractive option. Consequently, spectroscopic methods have been used to estimate almost all the properties previously discussed. Based on detergent fiber calibration, NIR has predicted biochemical composition of sugarcane (Sabatier et al., 2012), rice (Jin and Chen, 2007), corn stover and switchgrass (Liu et al., 2010), miscanthus (Hodgson et al., 2010) and several other species. Dietary fiber calibration has also been used to predict detailed monomeric sugar composition of corn (Wolfrum and Sluiter, 2009) and miscanthus (Hayes, 2012). Proximate and ultimate analysis and heating value have been estimated for rice straw using NIR (Huang et al., 2008, 2009). FTIR models have successfully been used to estimate N content, heating value and alkali index of switchgrass and reed canary grass (Allison et al., 2009), and lignin and heating value in poplar (Zhou et al., 2011). NIR has been used to estimate moisture, ash and heating value of spruce (Lestander and Rhén, 2005) as well as miscanthus and willow (Fagan et al., 2011) and heating value in sorghum and miscanthus (Roberts et al., 2011; Everard et al., 2012). Lestander et al., 2009 also show that NIR can even predict the energy required to pelletize sawdust, and NIR would likely have similar success in predicting the energy required to grind biomass. Though often omitted in methodological discussions, sample preparation can become the limiting step for any high-throughput phenotyping method. From this perspective, these may be less attractive due to necessary sample preparation steps. Both IR and NIR can utilize small sample sizes; <10 mg for IR and <100 mg for NIR (Laurens and Wolfrum, 2011) and while commonly the samples are ground, this is not always necessary (Penning et al., 2009). NIR is non-destructive, and through the use of various techniques [Attentuated Total Reflectance (ATR), Diffuse Reflectance (DR)], FTIR can also be non-destructive.

While research exploring the genetic basis for variation among these traits is often conducted in conditions that minimize the environmental variability (growth chambers and greenhouses), assessing the genetic and environmental interplay in field environments is essential to improving desirable traits in the new energy feedstocks. Recent efforts have begun to assess field populations with sensors that use various spectra of light and correlate with phenotypes such as plant height, biomass, drought tolerance and others (Montes et al., 2011; Normanly, 2012; White et al., 2012). These efforts need to be expanded to other bioenergy relevant traits. Non-destructive spectral imaging could be adapted from current applications in precision fertilizer application (Haboudane et al., 2002) to other compositional properties. These approaches might be used to predict heating value or even moisture content from spectral-based elemental composition (Seelig et al., 2008).

In summary, several primary analysis techniques might be amenable to high-throughput, mostly by automating the steps involved or multiplexing to process many samples at once. However there appears to be real promise in leveraging regression or multivariate approaches to predict key properties like heating value and ash based on data from elemental analyzers or spectral approaches.

## Conclusion

Thermochemical conversion of biomass is an increasingly viable way to use bioenergy crops and agricultural residues to fulfill energy needs. Plant biologists and engineers both have important roles to play in the design of thermochemical bioenergy systems that result in appropriate pairings of biomass feedstocks and conversion technologies, though each group is limited by the constraints of their respective sub-systems. For engineers, this involves the design of efficient supply chains and conversion technologies that are robust to natural variations in biomass properties while minimizing energy use, material costs, and harmful emissions. Preprocessing technologies such as baling straw (Lötjönen and Paappanen, 2013) or torrefaction (Robbins et al., 2012) can also contribute to feedstock standardization. For biologists, this involves optimizing favorable biomass traits without compromising the plant's ability to survive in a sometimes hostile ecosystem. Natural genetic variation is a powerful resource for the improvement of bioenergy traits (both enzymatic and thermochemical) in feedstock plants, and the biological community has made great progress in understanding and manipulating the genetic pathways behind various relevant plant traits. Conversely, if natural variation for a trait is low, it is likely that modifications would incur serious consequences for the plant.

We find sufficient variation in lignin and evidence for potential genetic manipulation and several relatively high-throughput measurement methods. Unlike enzymatic systems where lignin is highly problematic, its role is more nuanced in thermochemical conversion systems where it is associated with higher HHV feedstocks, but changes to product distributions that may or may not be desirable. From an agronomic standpoint, increased lignin might be more feasible than reduced lignin due to the important roles it plays in physical stability and protection against pathogens. While less clear how amenable ash content is to genetic manipulation, we find larger variation in ash and variation in many of the minerals that contribute to ash and suitable methods to phenotype them. Silica is a special problem with grasses, but could be addressed by exploiting the huge diversity observed within and among species, particularly using knowledge of the transporters with which its deposition might be controlled. Reducing silica in grasses and increasing lignin to mitigate the associated agronomic impacts might lead to an optimal thermochemical feedstock. However, the relationship between lignin, ash, and thermochemical conversion products is still not well understood, and additional systematic experimentation or meta-analysis will be necessary to confirm these strategies. The most biologically interesting traits may not be the traits that will have the greatest economic and lifecycle impact. Efforts to determine heritability in more abstract traits such as moisture content, grindability and bulk density would be valuable next steps based on observations of genetic variation seen in rice and other species, though high-throughput methods to measure grindability and density do not currently exist.

Biochemical and proximate/ultimate analysis are both equally valid paradigms for describing a kilogram of biomass, though the latter does present two distinct advantages in the context of feedstocks for thermochemical bioenergy production. Predicting biomass properties such as HHV from biochemical analysis results is challenging (likely because of biases associated with different measurement methods), while regressions based on ultimate analysis appears to work even across diverse data sets (Sheng and Azevedo, 2005). Secondly, ultimate analysis may prove to be more amenable to high-throughput phenotyping efforts, with automated elemental analyzers and spectroscopy as promising direct and indirect methods for the measurement of many important properties. While initial investments in equipment and model development can be high, the establishment of core facilities and modeling equations for thermochemical characterization of biomass can make these approaches more accessible.

While this review has focused on genetic approaches to crop improvement, agricultural management (including what fertilizers to apply, when to harvest, and how to store the biomass) is critical and will impact the characteristics of the biomass, and ultimately, the lifecycle of the system (Robbins et al., 2012; Davis et al., 2013; Wilson et al., 2013). Teasing out genetic variation and environmental effects has been and will continue to be a major challenge. Careful observation of all key traits, including agronomic traits related to sustainable crop production, will need to be done—some pathways are common to some molecules or elements, and plants may need to compensate for composition changes in unexpected ways. It is critical to analyze results in the context of the environment and avoid sweeping generalizations attributed to a certain species or specific transgenic plant (Voelker et al., 2010). Moving from the individual plant in the greenhouse to a field of plants will present new challenges and new surprises. Can we make valid conclusions from biomass composition at the field level, or will understanding genetic control require phenotyping at the resolution of individual plant organs or even cell types? Large-scale high-throughput phenotyping is the next frontier in plant science, and this review can help biologists and engineers prioritize traits for next generation bioenergy crop improvement. Beyond bioenergy, the food, forage, pulp, and paper industries will benefit as we fine-tune all aspects of biomass composition.

### Conflict of interest statement

The authors declare that the research was conducted in the absence of any commercial or financial relationships that could be construed as a potential conflict of interest.
